# Loss induced coherent combining in InP-Si_3_N_4_ hybrid platform

**DOI:** 10.1038/s41598-018-19280-7

**Published:** 2018-01-17

**Authors:** Yeyu Zhu, Yunsong Zhao, Lin Zhu

**Affiliations:** Department of Electrical and Computer Engineering, Center for Optical Materials Science and Engineering Technologies, Clemson University, Clemson, SC 29634 USA

## Abstract

Loss, as a time-reversed counterpart of gain, can also be used to control lasing in an optical system with coupled cavities. In this study, by manipulating mirror losses at different output ports of coupled Fabry-Perot cavities, an integrated coherently combined laser system is proposed and experimentally demonstrated in the InP-Si_3_N_4_ hybrid platform. Two InP-based reflective semiconductor amplifiers are coherently combined through an adiabatic 50:50 directional coupler in silicon nitride. The combining efficiency is ~92% at ~2× threshold. The novel system not only realizes the miniaturization of coherent laser beam combining but also provides a chip-scale platform to study the coherent coupling between coupled laser cavities.

## Introduction

It is well known that minimizing loss is important for a conventional laser cavity to improve the laser performance, i.e., reducing the lasing threshold and narrowing the spectral linewidth, since the loss must be compensated by gain. However, in an optical system with coupled cavities, the loss can be manipulated to provide novel functionalities. Recently, many counter-intuitive features have been observed by manipulating the loss in a coupled optical system^1^. For example, loss induced optical transparency is demonstrated in a system with coupled waveguides^2^. Through controlling the loss contrast, the unidirectional light propagation is obtained in the coupled silica microtoroids^3^. The onset of lasing in the coupled resonators is observed with the increased total loss in the system^4^. Although the loss is a natural adversary of lasing in most conventional laser cavities, we could control the optical field distribution in a coupled cavity by manipulating the loss, which offers a unique opportunity to create interesting laser devices. In this paper, we demonstrate that through introducing the extra mirror loss into the coupled Fabry-Perot (F-P) cavities in the hybrid InP-Si_3_N_4_ chipscale platform, a coherently combined laser system can be obtained without compromising the performance of the coupled lasers.

Hybrid integration has become a promising candidate for multi-functional photonic integrated circuits (PICs) beyond monolithic integration and has attracted intense interests in recent years^5–8^. In such a hybrid system, active and passive components are created respectively on their native substrates with suitable material systems^9–12^. The output of the active devices can be directly end-to-end coupled to the waveguides on the passive chip^13–15^. In this paper, two Indium phosphide (InP) gain chips, which provide the optical gain for the composite laser cavity, are coupled together through an adiabatic silicon nitride directional coupler, which provides the intercavity coupling, on a silicon chip. The cross-coupling results in the composite resonator modes (supermodes). Multiple supermodes are often supported in the coupled cavities and each supermode corresponds to a distinctive phase distribution among the gain elements^16^. Since the silicon platform is preferable for passive optical components due to the low cost and mature processing technology^17,18^, the tuning of cavity loss is obtained in the silicon chip. We control the cavity mirror loss by narrowing down the waveguide width to zero for one output port of the coupler, which effectively enhances the mirror loss for this output port. We show that a specific supermode can be selected through controlling the distribution of mirror losses at different output ports of the coupled cavities. Through the loss induced mode selection mechanism, we experimentally demonstrate coherent beam combining (CBC) in the chipscale hybrid platform and show that the additional loss does not compromise the performance of the coupled lasers due to the destructive interference at the lossy output port.

For a single semiconductor laser, it is difficult to obtain the high-power operation with good output beam quality due to the thermooptic/optical nonlinear effects. To scale up the output power, CBC can be used to overcome this limitation. However, the CBC laser system usually requires optical fibers^19,20^ or free-space components such as lens^21,22^, external cavities^21,23^, diffraction gratings^24,25^ and photonic crystals^26^. In our demonstration, we eliminate the need of fiber and free-space based optical components. Our miniaturized CBC laser system in the InP-Si_3_N_4_ hybrid platform not only is important for creating an integrated high-power laser source for passive PICs but also provides a chip-scale platform to study the phase locking dynamics in a laser system with coupled cavities.

## Laser design and fabrication

**Figure 1:**
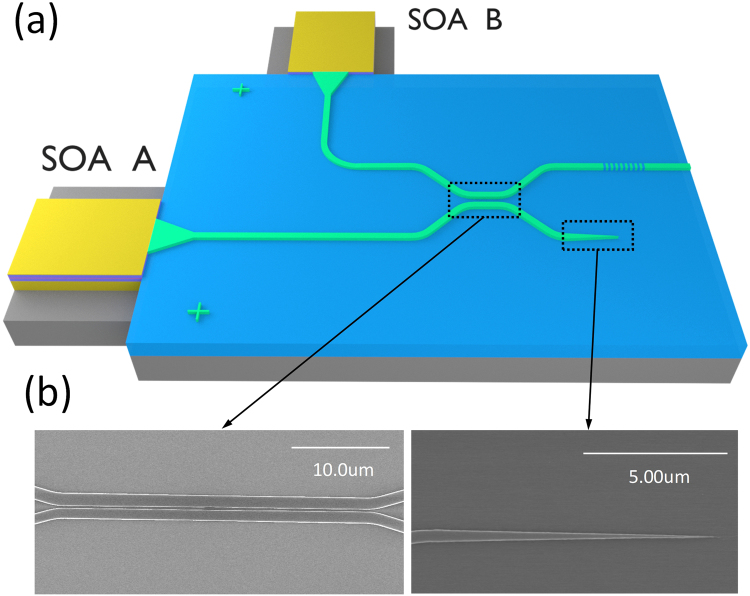
(**a**) Schematic plot of a hybridly integrated chipscale CBC laser system; (**b**) SEM images of the fabricated passive CBC cavity.

Figure 1(a) presents the schematic plot of the coherently combined laser system based on the hybrid integration approach. It consists of two InP-based reflective semiconductor optical amplifiers (SOAs), and a Si_3_N_4_/SiO_2_/Si chip where the SiO_2_ cladding layer is thick enough (4 *μm*) to prevent optical leakage from the Si_3_N_4_ waveguide layer to the Si substrate. The length of SOA is 1 *mm*. It has a high reflection (HR) coated back-facet with a 90% reflectivity. The SOA front facet is anti-reflection (AR) coated. The gain ripple of the SOA is less than 1 dB across a 40 *nm* range near 1550 *nm*. In the CBC cavity design, the two SOAs (A and B) are coupled by a 50:50 broadband directional coupler. The mode converter is used to improve the coupling efficiency between the gain chip and passive chip. One of the coupler output ports is made to be lossy by narrowing down the waveguide width as shown in Fig. 1. The other output port can provide optical feedback for lasing through a broad-band reflector, i.e., a DBR reflector or a cleaved facet. Coherent combining of the two SOAs is obtained by the extra mirror loss introduced at the output port. The width of the single mode silicon nitride waveguide is set to be 800 *nm* and the height is 300 *nm*. The gap between the waveguides at the coupling region is set to be 300 *nm*. Figure 1(b) also shows the SEM images of the fabricated CBC cavity.

The passive chip fabrication is summarized here. A 300-nm-thick Si_3_N_4_ is deposited on a SiO_2_-on-Si wafer using a Tystar Nitride LPCVD tool. To pattern the Si_3_N_4_ layer, a JEOL JBX-9300FS electron-beam lithography (EBL) system is used with ZEP520A (by Zeon cooperation) as the e-beam resist. Next, the pattern is transferred to the Si_3_N_4_ layer using plasma etching with a CF_4_/CHF_3_ gas mixture in an Oxford Endpoint reactive ion etching machine. After the resist is removed, a 2-μm-thick SiO_2_ is deposited on the Si_3_N_4_ layer using plasma-enhanced chemical vapor deposition process.

## Modal analysis of the CBC cavity

**Figure 2:**
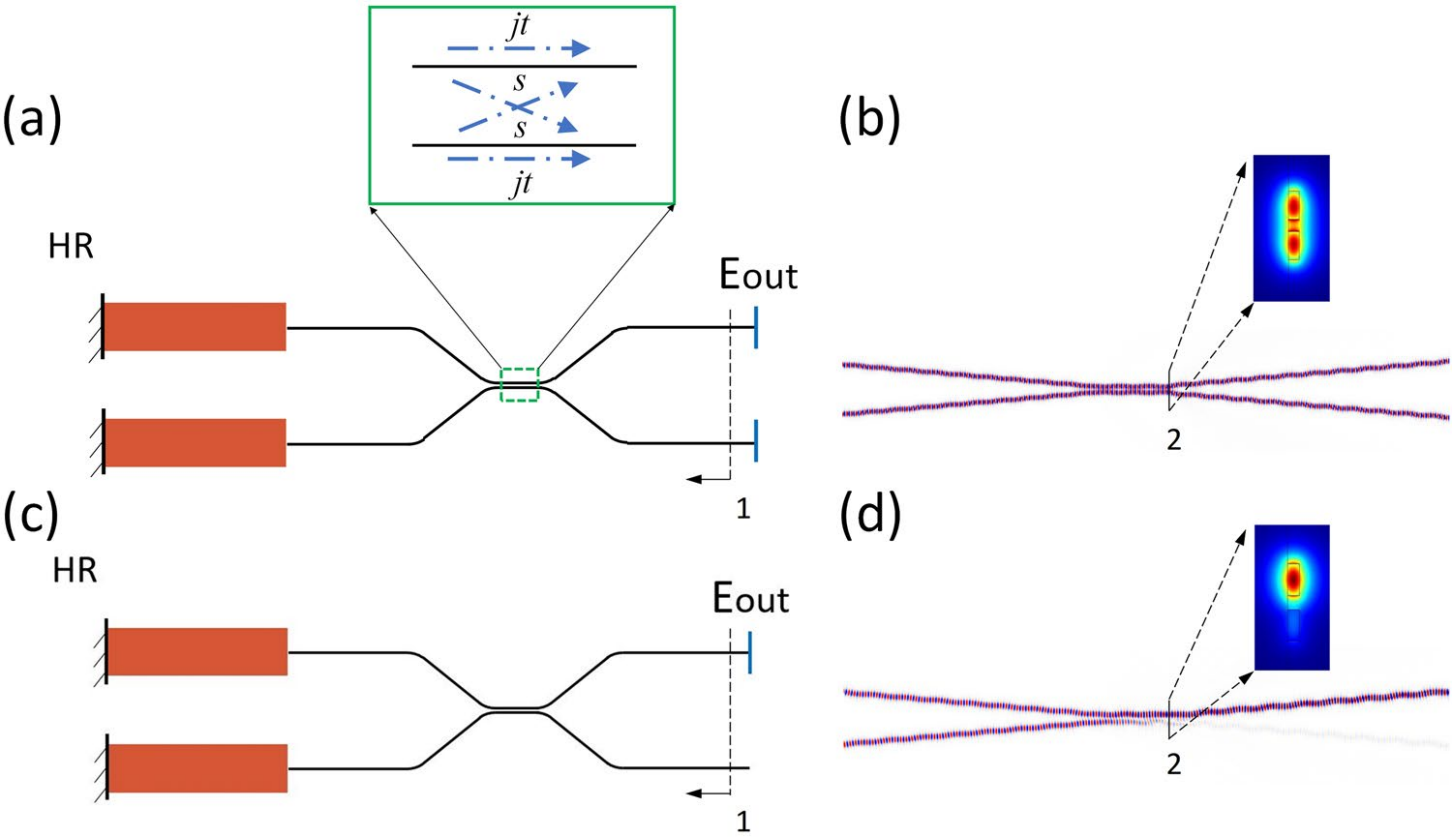
Schematic plot and FDTD/FEM simulation results of the combined laser cavities in the InP-Si_3_N_4_ platform without the extra loss (**a**,**b**) and with the extra loss (**c**,**d**).

The modal analysis of two types of coupled cavities in the InP-Si_3_N_4_ platform is performed by a transfer matrix method^27,28^. As shown in the schematic plot in Fig. 2(a) and (c), each system design consists of two gain chips (i.e., SOAs) at the left side and a 3-dB broadband coupler at the right side. The interface reflection between the gain element and coupler is assumed to be zero. Thus, each composite cavity includes two groups of end facets acting as the feedback mirrors: one is the left end facets of the gain elements and the other one is the right end facets of the coupler output ports. Figure 2(a) shows the combined laser cavity design without the extra loss added at one of the output ports, which means that the mirror losses at the two coupler output ports are the same (symmetric). Figure 2(c) shows the CBC cavity design where one of the coupler output ports is made to be lossy, so that the asymmetric mirror losses are introduced. Here, we assume that the two gain elements work at the same resonance frequency. The cavity supermodes can be obtained by solving the eigenvalue problem of the overall transfer matrix of the composite cavity. The transfer function of the 3-dB coupler shown in Fig. 2 is described by a matrix as:

1 $$[\begin{array}{cc}jt & s\\ s & jt\end{array}]$$

where *t* and *s* are described in the inset of Fig. 2(a), and they should satisfy |*t*|^2^ + |*s*|^2^ = 1 (*t* = *s* = $1/\sqrt{2}$ for a 3-dB coupler). Starting from position 1 (shown in Fig. 2(a) and (c)), the round-trip matrix for the light wave propagating in the cavity can be expressed as:

2 $$X={R}_{2}{A}^{\ast }S{R}_{1}SA$$

3 $${\rm{A}}=[\begin{array}{cc}{\rm{jt}} & {\rm{s}}\\ {\rm{s}} & {\rm{jt}}\end{array}]\,{\rm{S}}=[\begin{array}{cc}{{\rm{e}}}^{{\rm{j}}{{\rm{\varphi }}}_{1}} & 0\\ 0 & {{\rm{e}}}^{{\rm{j}}{{\rm{\varphi }}}_{2}}\end{array}]\,{{\rm{R}}}_{1}=[\begin{array}{cc}1 & 0\\ 0 & 1\end{array}]$$

4 $${R}_{2}=[\begin{array}{cc}r & 0\\ 0 & r\end{array}]\,{\rm{or}}\,{{R}}_{{\rm{2}}}=[\begin{array}{ll}r & 0\\ 0 & {r}_{t}\end{array}]$$

where *A* is the transfer matrix of the 3-dB coupler, *S* is the phase change in each gain element, *R1* and *R2* are the reflection matrices of the left and right end facets, and the operator * denotes the complex conjugate. In the following analysis, we assume the left end mirrors have perfect reflectivity. *r* is set to be 1/3 that is the estimated reflection coefficient at the interface between a Si_3_N_4_ waveguide and air. *r*_*t*_ is set to be 0.02 that is the estimated reflection coefficient at the lossy port. To support a supermode, the system should be able to reproduce the electric field *E* after one round trip, which means the following equation should be satisfied:

5 $$XE=\mu E$$

where *μ* is the eigenvalue of *X* and *E* is the eigenvector. Each pair of *μ* and *E* corresponds to a supermode supported by the coupled cavities. The eigenvector is the field distribution at the output ports and eigenvalue *μ* is the associated modal gain^27,28^. There are two elements in the eigenvector that correspond to the electric field amplitudes at the position 1 shown in Fig. 2. For the cavity without the extra loss shown in Fig. 2(a), if the phase change in the gain elements is the same, the nonzero eigenvalues and the corresponding eigenvectors are shown as follows:

6 $${\mu }_{a1}=1/3;\,|{E}_{a1}|=[\begin{array}{cc}1 & 0\end{array}]$$

7 $${\mu }_{b1}=1/3;\,|{E}_{b1}|=[\begin{array}{cc}0 & 1\end{array}]$$

Since the two eigenvalues are the same, the modal discrimination between the two eigenmodes is zero, which means the two supermodes will be induced at the same time. There will be the light emitting from both two output ports. This can also be demonstrated by the FDTD simulation results shown in Fig. 2(b). The inset shows the modal profile near the right end of the coupling region (position 2) through the FEM analysis (COMSOL). The optical field is evenly distributed at the two waveguides.

8 $${\mu }_{a2}=1/3;\,|{E}_{a2}|=[\begin{array}{cc}1 & 0\end{array}]$$

9 $${\mu }_{b2}=0.02;\,|{E}_{b2}|=[\begin{array}{cc}0 & 1\end{array}]$$

For the CBC cavity design in Fig. 2(c), the two eigenvalues are different, as shown in Eqs (8) and (9). The modal discrimination between the two eigenmodes becomes much larger. This means that only the supermode with the higher modal gain will be selected in the CBC cavity for lasing. The constructive interference occurs at the output port with the mirror feedback and the destructive interference occurs at the other output port. The supermode selection leads to the coherent combining of the two gain elements at the left side. This is also demonstrated by the FDTD analysis in Fig. 2(d). The FEM analysis in the inset shows the mode profile of the preferred lasing mode. Therefore, though there is an extra loss introduced into the laser cavity, the two SOA gain chips can be coherently combined without compromising the laser performance.

## Hybrid integration and measurement results

**Figure 3:**
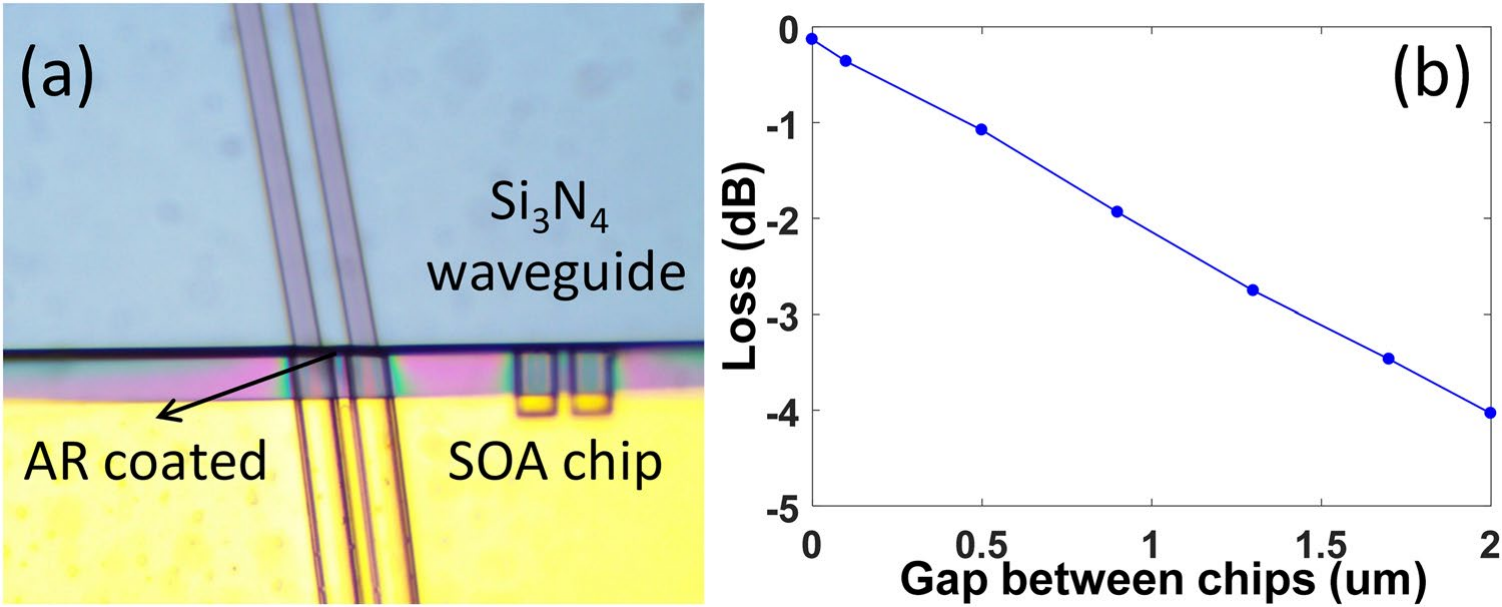
(**a**) A hybridly integrated SOA gain chip with a silicon nitride waveguide; (**b**) the insertion loss between the two chips as a function of the gap distance between them.

We use active alignment method to demonstrate the hybrid integration of two SOAs and a passive cavity^29^. The SOA is directly coupled to the passive waveguide through a spot-size converter. To eliminate the reflection at the interface between the SOA gain chip and the passive silicon chip, the ridge waveguide in the SOA gain chip and the Si_3_N_4_ waveguide are both angle-cleaved. Besides, the chip facet is AR coated. The interface reflectivity measured between the SOA gain chip and passive silicon chip is less than 0.01%. In Fig. 3(a), we show an optical image of a SOA gain chip directly coupled with a passive silicon chip. Figure 3(b) shows the variation of the insertion loss between the ridge waveguide in the SOA gain chip and the Si_3_N_4_ waveguide as a function of the gap distance between them. Since we use the cleaved facet for both chips, the smallest gap between the active chip and the passive chip we can obtain is ~1 *um* (the corresponding insertion loss is ~2.5 *dB*). The gap distance can be further reduced with a polished silicon chip facet.

**Figure 4:**
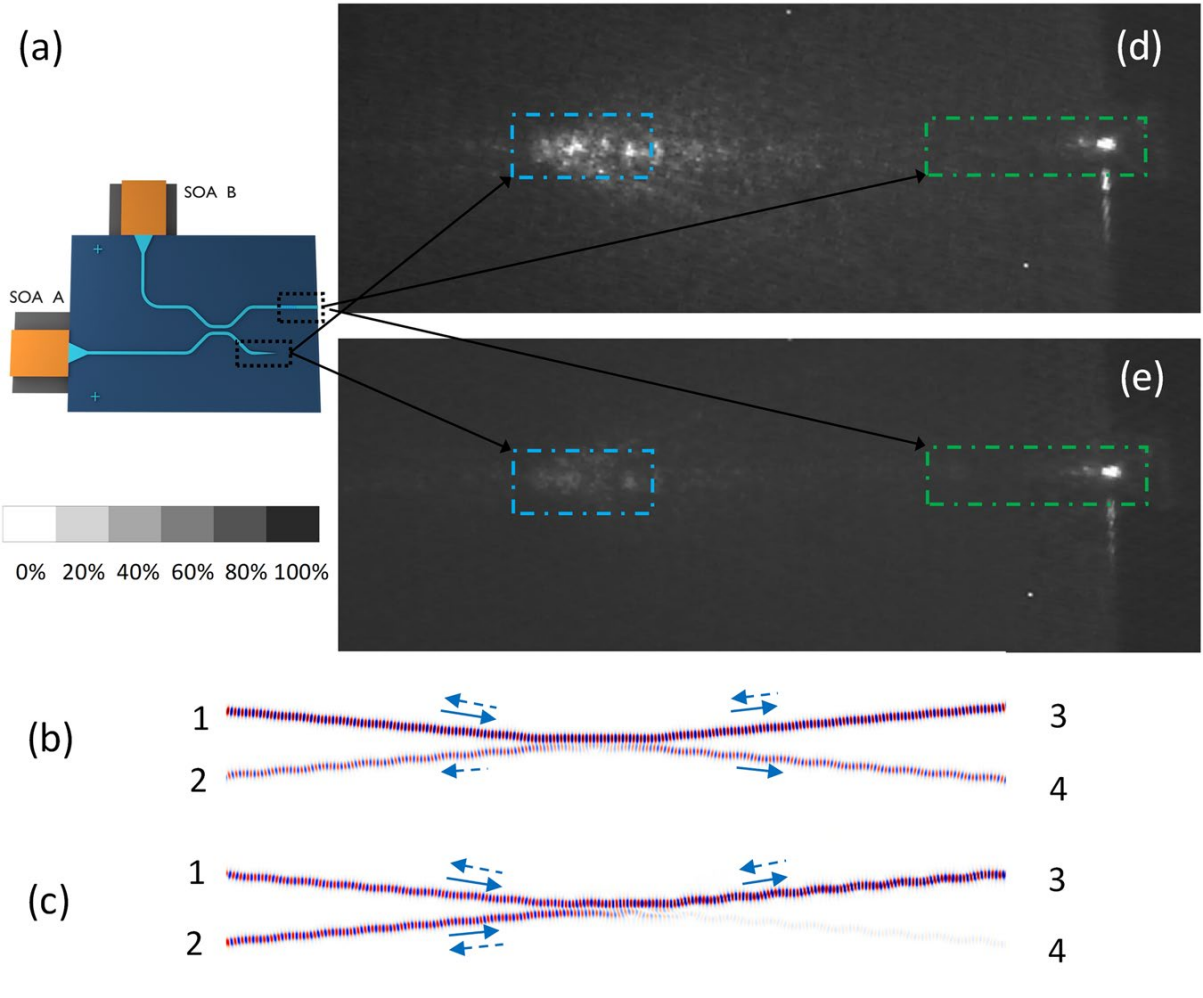
(**a**) Schematic plot of the hybridly integrated CBC laser system; FDTD simulation results and near field IR images (top-view) of the coherently combined lasers if only one SOA is turned on (**b**) and (**d**); both two SOAs are turned on at the same time (**c**) and (**e**). The grayscale bar shows the relative grey value.

Figure 4(a) shows the schematic plot of the hybridly integrated CBC laser system. If only one SOA (i.e., SOA B) is turned on, the emitted light will be equally coupled to two output ports of the coupler. Figure 4(b) shows the power flow at the coupling region through FDTD analysis. The light signal is injected from port 1 (SOA B), and is coupled into port 3 and 4 evenly. Then it is reflected back into the coupling region at port 3 through a broad-band reflector. Since SOA A is not turned on and port 4 is the lossy output port, the light transmitted to port 4 and 2 dissipates completely. The extra loss at these two output ports for one round trip is 6 dB. Figure 4(d) shows the top-view near field infrared (IR) image. The green line area corresponds to the Si_3_N_4_ waveguide with the cleaved facet, while the blue line area corresponds to the lossy output port of the coupler. As shown in the near field IR image, there is intense light scattered from the blue line area. Then, the two SOAs are turned on at the same time with the similar injection currents to optimize the combining efficiency. If the two SOAs are coherently combined and in-phase, they will constructively interfere at port 3 and destructively interfere at port 4 as shown in Fig. 4(c). This exactly matches our measurement results, as shown in Fig. 4(e). From the near field IR image, we can find that the intensity of the scattered light from the lossy output port of the coupler is greatly suppressed, while the output power obtained at the cleaved facet remains the same with the single SOA operation. The ratio between the brightness of port 4 in Fig. 4(d) and the one in Fig. 4(e) is ~9. The results in Fig. 4 demonstrate that the two lasers are coherently combined through the passive CBC cavity.

**Figure 5:**
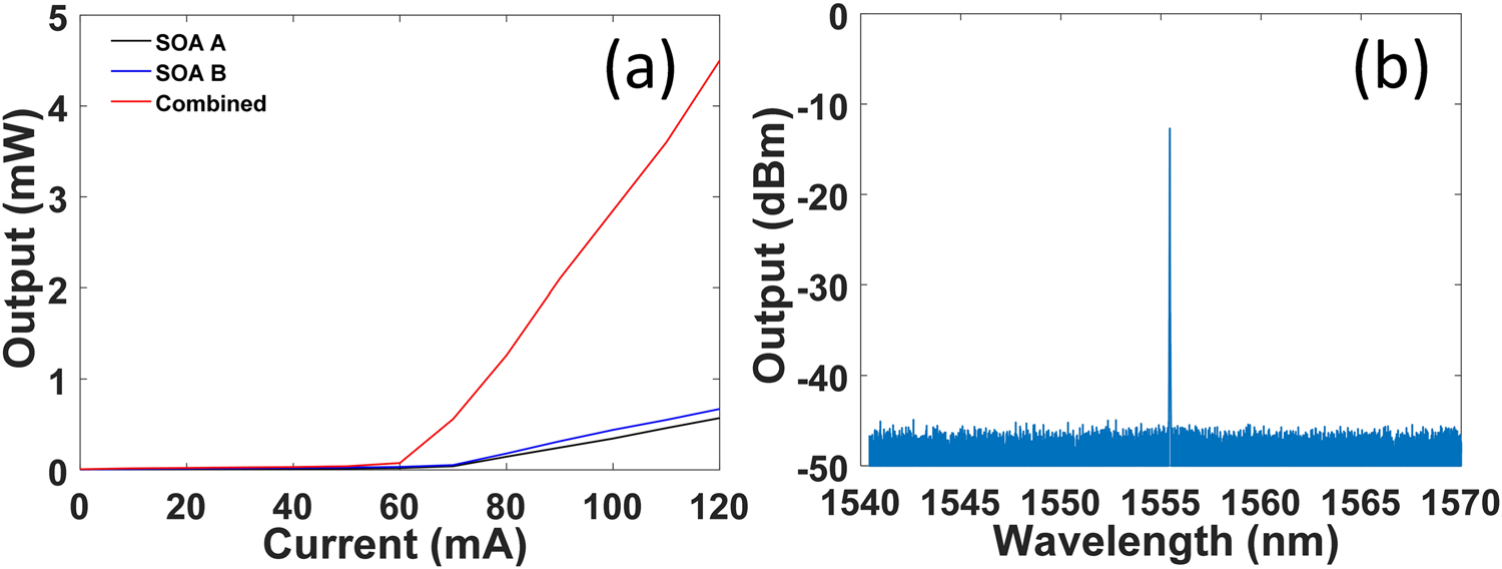
LI curves (**a**) and optical spectrum (**b**) of the coherently combined laser system.

Figure 5(a) shows the LI curves of the two coherently combined lasers and the single individual laser. For the single individual laser operation, we only turn on one SOA. The corresponding LI curves are shown by the black or the blue line. For the coherently combined lasers, both SOAs are turned on at the same time. The corresponding LI curve is shown by the red line. Compared with the coherently combined lasers, there is an additional 6 dB loss for the single individual laser, as we discussed before. Therefore, with the 100% combining efficiency, the output power of the coherently combined lasers should be eight times the output power of the single individual laser if the pump currents are set to be the same. From Fig. 5(a), we can find that the combining efficiency of the combined lasers is ~92% at I = 120 mA (~2× threshold). The measured high combining efficiency also proves that the two SOA are indeed coherently combined. Figure 5(b) shows the optical spectrum for the combined lasers. The pump currents are set at 100 mA. The output power of our combined lasers is currently limited by the maximum injection current allowed for the SOAs. In addition, we have to mention that it is difficult to obtain two single laser cavities with the same optical lengths. In our experiments, we firstly set the same injection currents in each SOA to obtain the similar optical powers in each laser cavity. Then, in order to obtain the optimal combining efficiency, the injection current in one of the two SOAs will be tuned slightly, which can help us find the common resonant mode and the phase locking condition. But the injection current difference between the two SOAs is always small, usually less than 1 mA.

For the traditional evanescently-coupled laser array, all the supermodes have the similar modal gains so that there is little modal discrimination among them. It is important to enhance the modal discrimination for the efficient and robust single-mode operation. Here, we improve the modal discrimination and performance of the coherent array by introducing the extra mirror losses into the coupled cavities. Normally loss will result in energy dissipation that is ubiquitous in nature. However, by taking advantage of the different field distributions induced by the extra loss, we demonstrate the coherent beam combing without compromising the laser performance.

**Figure 6:**
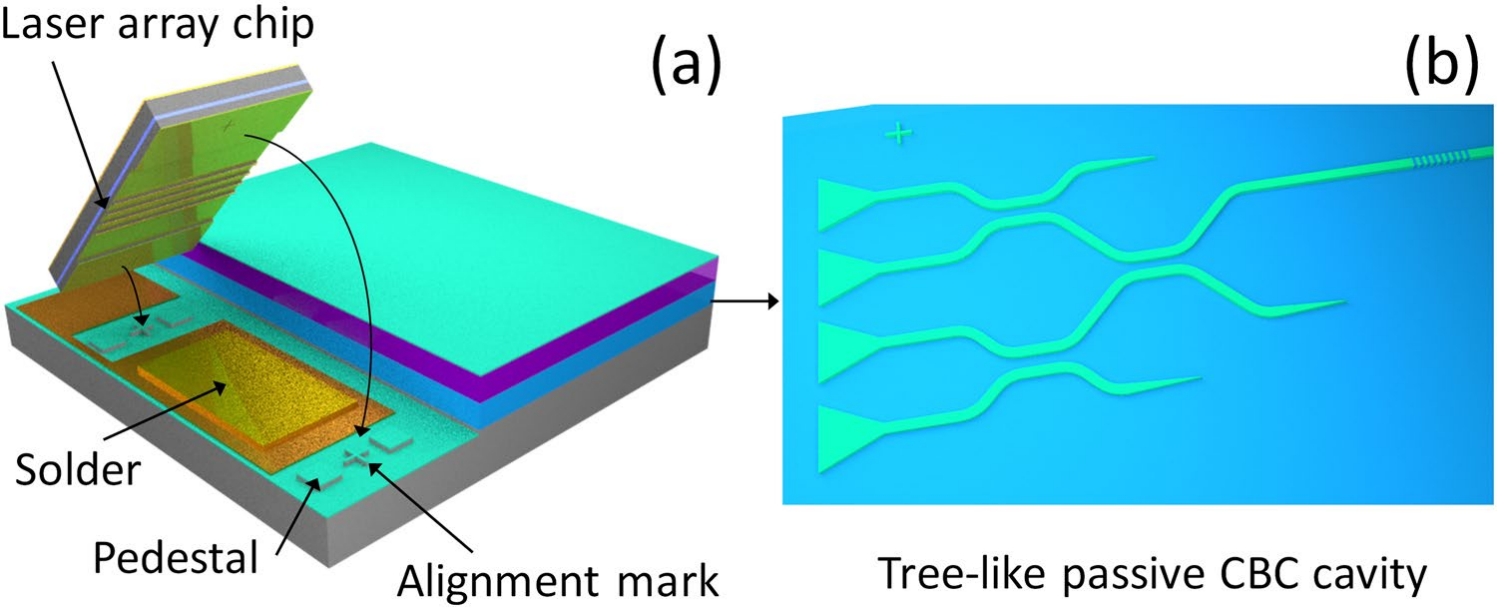
Schematic plot of a fully integrated CBC laser system.

Although only two SOA gain chips are coherently combined through active alignment in this paper, the demonstrated approach can be expanded to a fully integrated coherently combined laser array system as shown in Fig. 6^30,31^. Figure 6(a) shows the schematic plot of a hybrid integration procedure where a laser gain chip is flip-chip bonded on a silicon chip. Through the hybrid integration, the vertical position of the laser chip is entirely determined by the silicon pedestals and III-V insulation layers. Figure 6(b) show the schematic plot of the passive CBC cavity for four emitters. The proposed hybrid CBC laser system is important for realizing on-chip high power laser sources for integrated nonlinear optics applications, such as micro-resonator based optical frequency comb generation, on a single silicon chip.

## Conclusion

We experimentally demonstrate the loss induced coherent combining of two SOAs in an InP-Si_3_N_4_ hybrid platform. The CBC is realized by controlling the mirror loss distribution of the coupled cavities, due to the tendency of the lasing to self-adaptively operate with minimum losses. From the measurement results, we show that the combining efficiency of our system is ~92% at ~2× threshold. Besides, we propose a fully integrated coherently combined laser array system on a single chip. The diode laser based chip-scale CBC system provides the desired system compactness by removing the need of external free-space/fiber components and coupling optics. In addition, it is an ideal platform for investigating the coherence coupling between different lasers.
